# Up-regulated lncRNA XIST contributes to progression of cervical cancer via regulating miR-140-5p and *ORC1*

**DOI:** 10.1186/s12935-019-0744-y

**Published:** 2019-02-28

**Authors:** Xing Chen, Dongsheng Xiong, Liya Ye, Kai Wang, Lingfei Huang, Shuangshuang Mei, Jinhong Wu, Shanshan Chen, Xiaoli Lai, Lingzhi Zheng, Meifen Wang

**Affiliations:** 1 Department of Obstetrics and Gynecology, Taizhou Hospital of Zhejiang Province, Wenzhou Medical University, No. 150 Ximen Street, Linhai, 317000 Zhejiang China; 2 Center for Uterine Cancer Diagnosis & Therapy Research of Zhejiang Province, Hangzhou, 310000 Zhejiang China; 3 State Key Laboratory of Experimental Hematology, Institute of Hematology & Hospital of Blood Disease, Chinese Academy of Medical Sciences & Peking Union Medical College, Tianjin, 300020 China

**Keywords:** XIST, miR-140-5p, *ORC1*, Cervical cancer cells

## Abstract

**Background:**

The study purpose was to make investigation into the influence of XIST on cervical cancer progression and what’s more its potential mechanism.

**Methods:**

The cervical cancer data sets (lncRNA, miRNA, and mRNA) obtained from TCGA were analyzed with the “mixOmics” R package. Then, the expression of XIST, miR-140-5p, and *ORC1* were detected using qRT-PCR and western blot in both tissues and cervical cancer cell lines (Hela and C33A) to verify the bioinformatics analyses results. CCK-8 assay, 5-ethynyl-2′-deoxyuridine (EdU) assays, cell cycle assay and cell apoptosis assay were practiced. Besides, immunohistochemistry staining was operated for the detection of the Ki-67, E-cadherin and vimentin expression in cervical cancer tissues and the apoptosis-related proteins expression (c-caspase3, Bcl-2, total PARP and cleaved PARP) was verified through western blot. And in vivo experiments were implemented.

**Results:**

MiR-140-5p was down-regulated but XIST and *ORC1* were up-regulated in cervical cancer tissues and cell lines. Knocking down of the XIST or *ORC1* memorably suppressed cell proliferation, blocked cell cycle, decreased the expression of Bcl-2 while increased the apoptosis rate and the expression of c-caspase3 and cleaved PARP in HeLa and C33A cells. Besides, the results of immunohistochemistry staining showed knocking down the expression of XIST improved the expression levels of E-cadherin and decreased Ki-67 and vimentin expression. And overexpression of miR-140-5p also could inhibit the progression and reverse the influence of XIST and *ORC1* in HeLa and C33A cells.

**Conclusion:**

Our study indicated the effects of XIST/miR-140-5p/*ORC1* axis on the progression of cervical cancer which will shed new light on epigenetic diagnostics and therapeutics in cervical cancer.

## Background

Cervical cancer is among the most popular malignant tumors in women, and it tends to occur in young patients, in particular in the low and middle-income countries. The pathogenesis for cervical cancer is various, including the infection of human papiloma virus (HVP), herpes simplex virus type 2 (HSV-2), cervical thymus (CT) and other unhealthy living habits [1]. In recent years, many therapeutic methods have been developed, like surgery operation, radiation oncology and chemical treatment, but the 5-year survival rate for advanced patients is still very low [2, 3]. After the targeting interaction between long non-coding RNAs (lncRNA), messenger RNAs (mRNA) and microRNA (miRNA) being found, more biological medicine researches and treatments turns to gene therapy field, and many investigations related with the regulations between various types of RNAs and proteins in cancer cells have been undertaken. Hence, we aimed to find the mechanism of specific lncRNA targeting miRNA and mRNA in cervical cancer cells.

R language is an effective system in biology analysis, and mixOmics is an essential package which enables large-scale biological data sets combine together and run at the same time [4]. MixOmics analysis could be applied in exploring the biological data set by means of multivariate analysis, evaluating the molecular interactions happened at multiple levels in function and acquiring a large scale of information simultaneously [5]. Therefore, mixOmics can provide information that is obtained in a holistic manner and contribute to the biological development [5]. Here, we used mixOmics to integrate different data sets and attempted to reveal the potential molecular mechanism of cervical cancer.

The newly identified lncRNAs are members of the non-coding RNA family, whose length larger than 200 nucleotides and lacking protein-coding ability [6]. It has been demonstrated that lncRNAs play a significant role in many cancers progression and numerous kinds of cellular biological and pathological processes including invasion, apoptosis, proliferation, differentiation, epithelial mesenchymal transition (EMT) together with inflammation [7], like Homeobox (HOX) transcript antisense RNA (HOTAIR), brain cytoplasmic RNA 1 (BCYRN1) and small nucleolar RNA host gene 20 (SNHG20) [8–10]. Another important lncRNA of human is X-inactive specific transcript (XIST). XIST is the first non-coding gene identified within the region of chromosome X inactivation center (XIC), which silences one of the pairs of X chromosomes during the early developmental process in mammalian females [11]. It also tends to play a potential role as novel predictor of human cancer prognosis and is an abnormally expressed lncRNA in several cancers, which negatively related to clinical outcome [12]. Up to now, XIST was certificated to participate in the migration, growth and invasion of cancer cells and influenced the occurrence as well as development of cancers [13–15]. It was also proved that the XIST expression was correlated with the survival rate on the whole in patients who have cervical cancer, while the regulation process in the cancer cells have not been discussed yet [16]. Thus, we wanted to know more about the XIST’s effects on cervical cancer cells remains unclear.

MiRNAs, the short non-protein coding RNAs, have been proverbially reported to be important during the pathogenesis of multiple human cancers. A study indicated that the downregulation of miR-135b suppressed gastric cancer cells proliferation as well as the CDDP resistance of gastric cancer cells and induced cell apoptosis [17]. Wang et al. indicated that inhibition of miR-100 expression could reduce breast cancer metastasis [17]. MiR-221-3p from cervical squamous cell carcinoma (CSCC)-secreted exosomal could promote lymphangiogenesis and lymphatic metastasis after being transferred into human lymphatic endothelial cells (HLECs) [18]. Moreover, there are also some studies focusing on the function of miRNAs on cervical cancer. Poudyal et al. [19] demonstrated that overexpression of miR-6852 could lead to arrest the cell cycle in G2/M phase and induce necrosis in cervical cancer cell lines. In Su et al.’s study, it turned out that the expression of miR-140-5p was closely related to cervical tumor growth and metastasis [20]. Another study demonstrated that miR-140-5p expression was decreased in cervical cancel [8]. Also, one common point of XIST’s function in all these cancer cells lines on its regulation towards different kinds of miRNAs. Early studies indicated that XIST interacted with miR-140-5p to control lung cancer growth [4], as well as enhanced pancreatic carcinoma development [21]. Thus, we suspected that miR-140-5p was the potential target of XST1, and might also play important roles in cervical cancer.

*ORC1* is one type of origin recognition complex (ORC) gene whose location changes during cell cycle and is regulated during the cell division cycle, and it is very important in the initiation of DNA replication [22]. It was reported that *ORC1* is synthesized during G1 and degraded as the cell moves through the S phase, while the expression change of the other ORC subunits was not observed in a cell cycle-dependent manner [23]. As there have been many studies confirmed that *ORC1* was a key factor in cells cycle control, we were more interested in whether it can also regulate cell apoptosis.

Although XIST is concerned with the survival rate in cervical cancer patients, the exact modulating mechanism and the impacts of XIST on cancer cells are still worth to be further studied. We designed and conducted experiments in vitro and in vivo for understanding the XST1 function on the development of cervical cancer along with the regulating mechanism through miR-140-5p/*ORC1*.

## Methods

### The cancer genome atlas (TCGA) dataset

The cervical squamous cell carcinoma and endocervical adenocarcinoma (CESC) datasets were got from the TCGA online database (http://cancergenome.nih.gov/). The raw data of the tumor tissues and matched normal tissues were corrected and normalized by “DESeq2” and “edgeR” package using R software (version 3.4.1). For analyzing the differentially expressed genes (DEGs), the threshold of *P* value (after being adjusted by Benjamini and Hochberg method) was under 0.05 level of the Wald test, and the threshold of log2 (fold change) was > 1. Then the differentially expressed lncRNAs, miRNAs, and mRNAs were used for multivariate analysis with “mixOmics” package.

### Multivariate analyses using mixOmics package

The R package “mixOmics” was implemented to finish multivariate analysis in the biological data sets, and multiple functions such as data exploration, dimension reduction and visualization. According to provider’s instructions (www.mixOmics.org, [5]), the DEGs data were input into the R 3.4.1 software for Stacked Partial Least-Squares Discriminant Analysis (SPLSDA). Afterwards, analysis of the first component was carried out in order to obtain relevance network (r = 0.7). A circos plot was yielded for exhibiting the selected features within different types in a circle. The connections between or within omics were representatives of strong positive or negative correlations. Starbase (http://starbase.sysu.edu.cn) was practiced in predicting target among the first components.

### Cell culture

Cervical cancer cell lines (CaSki, HeLa, C33A, SiHa), human cervical epithelial cell line HcerEpic and human embryonic kidney cell line 293T were got from BeNa Culture Collection (Beijing, China). The cell lines CaSki and HeLa were maintained in 90% Roswell Park Memorial Institute (RPMI)-1640 with 10% fetal bovine serum (FBS). The cell lines C33A and HcerEpic were maintained in 90% Eagle’s minimum essential medium (EMEM) with 10% FBS. The cell line SiHa was maintained in minimum essential medium-Earle’s balanced salts (MEM-EBSS) with 10% FBS. All the cell lines were maintained at 37 °C in humid air with 5% CO_2_.

### Tissue samples collection

The 30 paired non-tumor adjacent tissue samples [the closest from the tumor (> 5 cm)] and cervical cancer tissue samples used in this study were collected from 30 patients who were diagnosed as cervical cancer and had undergone surgery at Taizhou Hospital of Zhejiang Province between 2014 and 2016. No patients received treatment before the operation. All the samples were collected, fixed with formalin and embedded by paraffin in conformity to standard methods for the following experiments. The research was ratified by the Research Ethics Committee of Taizhou Hospital of Zhejiang Province. The informed written consent was received from each participant. The clinical information was shown in Table 1.

**Table 1:** Correlation between expression of lncRNA XIST and clinic pathological features in cervical cancer patients (n = 30)

Parameters	Group	Total	lncRNA XIST^#^		*P*
			High	Low	
Age	≤ 60	22	11	11	0.544
	> 60	8	3	5	
Tumor size	≤ 5 cm	22	16	6	0.034*
	> 5 cm	8	2	6	
Figo stage	I	15	4	11	0.033*
	II	7	6	1	
	III	4	3	1	
	IV	4	1	3	
Tumor differentiation	Well	7	6	1	0.027*
	Moderate	5	3	2	
	Poor	18	5	13	
Pathological type	Squamous	14	8	6	0.746
	Adenocarcinoma	16	6	10	
Lymphatic metastasis	Yes	20	12	8	0.038*
	No	10	2	8	

^#^Low and high expression group were divided according to the median ratio of relative lncRNA XIST expression

* *P* value was determined by chi-square analysis. *P *< 0.05 was considered statistically significant

### Quantitative real-time PCR

Total RNA was isolated from the tissues or the cell lines by means of TRIzol reagent (Invitrogen, Carlsbad, CA, USA) in line with the supplier’s instructions. Then, the reverse transcription was done with a Reverse Transcription Kit (Takara, Tokyo, Japan). Real-time PCR analysis was practiced with SYBR Green (Takara). The amounts of expression level were numbered by the 2^−ΔΔ*Ct*^ method and the relevant expression levels were in normalization to GAPDH expression. QRT-PCR reactions were performed by the ABI7500 system (Applied Biosystems, Shanghai, China). The primer sequences were synthesized from Sangon Biotech and listed in Table 2.

**Table 2:** Primer sequences for qRT-PCR

Gene	Sequences
XIST	
Forward sequence	5′-AATGACTGACCACTGCTGGG-3′
Reverse sequence	5′-GTGTAGGTGGTTCCCCAAGG-3′
hsa-miR-140-5p	
Forward sequence	5′-CAGUGGUUUUACCCU-3′
Reverse sequence	5′-TGGTGTCGTGGAGTCG-3′
ORC1	
Forward sequence	5′-GTCCAATGTTGTAGCCGTGC-3′
Reverse sequence	5′-CGACGCTGAGATGGGATTGT-3′
GAPDH	
Forward sequence	5′-CAAGGTCATCCATGACAACTTTG-3′
Reverse sequence	5′-GTCCACCACCCTGTTGCTGTAG-3′
U6	
Forward sequence	5′-CTCGCTTCGGCAGCACA-3′
Reverse sequence	5′-AACGCTTCACGAATTTGCGT-3′

### Cell transfection

XIST sequence was amplified by the implementation of PCR, and then cloned into the *Xho*I and *Kpn*I sites of pcDNA3.1 vector to overexpress XIST. The miR-140-5p mimics and miR-140-5p inhibitor were obtained from RiboBio (Guangzhou, China). The small interfering RNAs (siRNAs) specifically targeting XIST and *ORC1* were designed and synthetize by Sangon Biotech (Shanghai, China). According to the manufacturer’s indication, two cell lines (Hela and C33A) were transfected with pcDNA3.1-XIST plasmid, miR-140-5p mimics/inhibitors and siRNA oligonucleotides with Lipofectamine 2000 (Invitrogen). The siRNA sequences were shown as follow:

Si-XIST-1 sense: 5′-CCAUGCACCUUGGACAUAA-3′;

Si-XIST-1 antisense: 5′-UUAUGUCCAAGGUGCAUGG-3′;

Si-XIST-2 sense: 5′-GCUUCUAACUAGCCUGAAU-3′;

Si-XIST-2 antisense: 5′-AUUCAGGCUAGUUAGAAGC-3′;

Si-*ORC1*-1 sense: 5′-CCAUGCACCUUGGACAUAA-3′;

Si-*ORC1*-1 antisense: 5′-CCAUGCACCUUGGACAUAA-3′;

Si-*ORC1*-2 sense: 5′-GCTGGAGCTTGGCAACTTA-3′;

Si-*ORC1*-2 antisense: 5′-UAAGUUGCCAAGCUCCAGC-3′.

### Luciferase reporter assay

PCR was conducted to amplify the fragment from XIST which contained the predicted miR-140-5p binding site, then the fragment was cloned into a pmirGLO Expression Vector (Promega, Madison, WI, USA) to form the XIST-wild-type reporter vector (XIST-wt). The miR-140-5p seed sequence binding site was mutated to create the corresponding mutant which was named as XIST-mut. 293T cells were co-transfected with miR-140-5p mimics or NC, and XIST-wt or XIST-mut plasmids using Lipofectamine 2000 (Invitrogen). For testing the relation between miR-140-5p and *ORC1*, the 3′-UTR region of *ORC1* containing the binding site of miR-140-5p was constructed into the pGL3 vector (Promega). The cells were transfected with miR-140-5p mimics or NC, and *ORC1*-wt or *ORC1*-mut plasmids. The Dual-Luciferase Reporter Assay System (Promega) was used for conducting luciferase reporter assay.

### RNA pull-down by MS2-MBP

Maltose-binding protein (MBP)-affinity purification was practiced for identifying the XIST-associated miRNAs. The MS2-MBP protein was in expression and purity from *E. coli* following the protocol of the Steitz laboratory. Three bacteriophage MS2 coat protein-binding sites (5′-CGTACACCATCAGGGTACGAGCTAGCCCATGGCGTACACCATCAGGGTACGACTAGTAGATCTCGTACACCATCAGGGTACG-3′) were inserted in the downstream of XIST with site-directed mutagenesis by the Stratagene Quik Change Site Directed Mutagenesis Kit. To acquire miRNAs relevant with MS2-tagged XIST, the cell lines Hela and C33A were in transfection with MS2-tagged XIST constructs. Each immunoprecipitation assay cost one million cells. 48 h after the transfection, the cells went through RNA pull-down analysis [24].

### CCK-8 assay

Cell proliferation was measured by the cell proliferation reagent CCK-8 (Roche, Basel, Switzerland). After the cells (1 × 10^3^/well) were plating in the 96-well microtiter plates (Corning, NY, USA), 10 μL CCK-8 regents were added to each well at the time of harvest. 2 h later, the absorbance was recorded at 450 nm to determine the cell viability.

### 5-Ethynyl-2′-deoxyuridine (EdU) assays

Besides the CCK-8 assay, we performed EdU assay to detect cell proliferation using the EdU DNA Proliferation in vitro Detection kit (RiboBio, China) according to manufacturer instructions. Cells were maintained into 96-well plate. 48 h after transfection, the cells were stained by EdU and DAPI. Proliferating rates were calculated using the ratio of the fluorescent positive cells to total cells.

### Cell cycle analysis

The Cell Cycle Detection Kit (Beckman Coulter, Brea, CA) was used for cell cycle analysis. The transfected cervical cancer cells were washed with PBS after being trypsinized, fixed in 70% ethanol at 4 °C overnight, stained with 20 mg/mL of propidium iodide (PI) at 37 °C for 30 min, and the cell cycle stage was then analyzed using the flow cytometer.

### Cell apoptosis analysis

Cells after transfection were harvested and stained by propidium iodide (PI) lasting 30 min. The FITC-Annexin V Apoptosis Detection Kit (BD Biosciences) based on the double staining by FITC-Annexin V and PI was applied in detecting the apoptosis level. Then, the apoptotic cells were analyzed through a FC500 flow cytometry with a Cell Quest 3.0 software.

### Western blot

Using of RIPA protein extraction reagent (Beyotime, Shanghai, China) with PMSF (Roche, Basel, Switzerland) to lyse cells. Approximately 25 μg of protein extracts were separated with 10% sodium dodecyl sulfate polyacrylamide gel electrophoresis (SDS-PAGE), transferred onto nitrocellulose membranes (Sigma, St. Louis, MO, USA), and probed with primary antibodies (anti-ORC1, ab85830, 1:5000; anti-actived-caspase3, ab2302, 1 µg/mL; anti-Bcl-2, ab692, 1:500; GAPDH, ab9485, 1:2500). Subsequently, secondary antibodies were provided to culture the cells (anti-Rabbit IgG H&L, ab6721, 1:10,000; anti-Mouse IgG H&L, ab205719, 1:10,000; Abcam). Membranes were in incubation at 4 °C for 24 h, then with secondary antibodies for another 2 h. The bands were detected by enhanced chemiluminescent (ECL) method. All antibodies were brought from Abcam (Cambridge, MA, USA).

### Xenograft assay

Five-week-old female athymic BALB/c mice were raised in specific conditions with pathogen-free and manipulated in line with protocols authorized by the animal center of Taizhou Hospital of Zhejiang Province. Mice were randomly divided into 2 groups (n = 4/group): Control group and si-XIST group. The cell line Hela transfected with RNAi vector was harvested at a concentration of 2 × 10^7^ cells/mL. 0.1 mL of the suspend cells was then injected in two sides of the posterior flank of nude mice. Tumor volumes (π/6 × minor axis^2^ × major axis) were inspected every 7 days as the implantations began to develop bigger. All mice were killed after 7 weeks of injection and the tumors were excised, weighed and paraffin embedded.

### Immunohistochemical analysis

A heat-induced technique was practiced to deparaffinize, rehydrate, and treat the paraffin-embedded tissues by 0.3% hydrogen peroxide for antigen retrieval. Then for staining, the samples were treated with Ki-67, E-cadherin and vimentin antibodies (Abcam, Cambridge MA) and background sniper (Biocare Medical, Concord, CA). MACH 4 Universal HRP Polymer detection kit (Biocare Medical) and 3,39-diaminobenzidine (DAB substrate kit, Vector Laboratories, Burlingame, CA) were used for measuring the proteins expression. The hematoxylin, dehydrated, mounted with VectaMount (Vector Laboratories) were employed to counter stain the slides, and Olympus BX 41 Microscope (Olympus Corporation, Japan) for visualization.

### Statistical analysis

All the data were expressed as mean ± SD (standard deviation) and the statistical analyses were practiced by GraphPad Prism (Version 7.0, La Jolla, CA, USA). Two-side student’s t-test, χ^2^ test or Wilcoxon test were conducted to measure the differences between groups. *P* < 0.05 was considered to have statistical significance.

## Results

### Bioinformatics analyses with mixOmics package

**Fig. 1:**
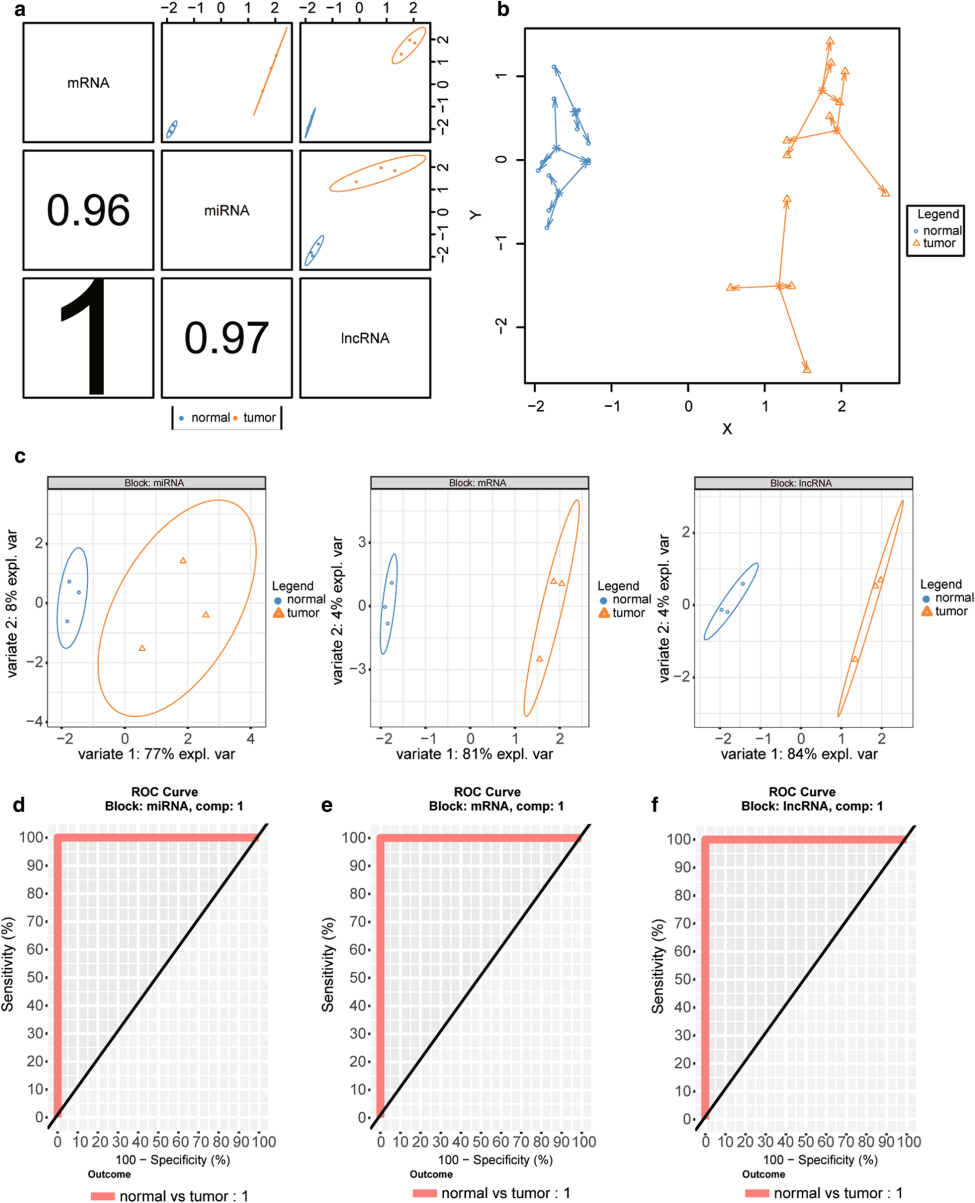
Partial least squares discrimination analysis with DIABLO. **a** Sample scatterplot from plotDiablo displaying the first component in each data set (upper diagonal plot) and Pearson correlation between each component (lower diagonal plot). **b** Arrow plot representing each sample pointing towards its outcome category. **c** The PLS-DA sample plots. **d**–**f** The ROC curve for miRNA (**d**), mRNA (**e**), and lncRNA (**f**) respectively

**Fig. 2:**
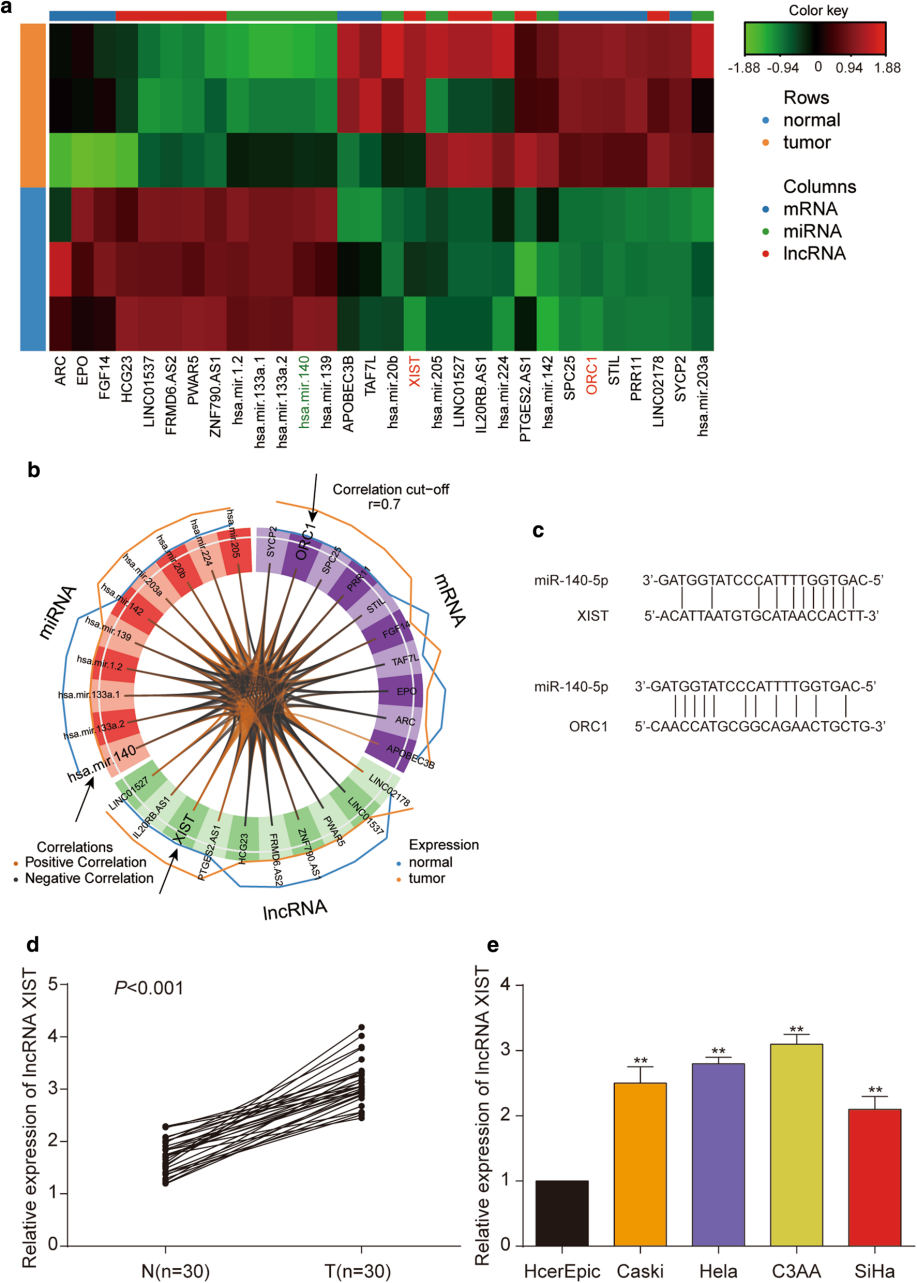
DIABLO graphical outputs on the cervical cancer study. **a** Clustered Image Map (Euclidean distance, Complete linkage) of the multi-omics signature. Samples are represented in rows, selected features on the first component in columns. **b** Circos plot shows the positive (negative) correlation (r = 0.7) between selected features as indicated by the brown (black) links, feature names appear in the quadrants. **c** The target relationships between XIST and miR-140-5p as well as miR-140-5p and *ORC1* were predicted by starBase. **d** The expression of XIST was determined by qRT-PCR in 30 pairs of cervical cancer tissues (T) compared with adjacent non-tumor tissues (N), which was verified statistical significance by *t*-test. “n” indicates sample number. **e** The expression of XIST was examined by qRT-PCR in four cervical cancer cell lines (CaSki, HeLa, C3AA, SiHa) and human cervical epithelial cell line HcerEpic, **P < 0.01, compared with HcerEpic cell line

In present study, we firstly analyzed the differentially expressed lncRNA, miRNA and mRNA between matched normal adjacent samples and tumor (CESC) samples. Then, the similarities between tumor samples and normal samples were assessed by using the SPLSDA method. Figure 1a showed that the first components from each data set were highly correlated to each other. In Fig. 1b, c, the first SPLSDA component distinguished CESC samples by normal samples with efficiency, but with the second SPLSDA component, separation between tumor samples and normal samples were small. Therefore, the subsequent bioinformatic analyses were on the basis of the first SPLSDA component. In this study, we used the receiver operating characteristic curve (ROC) assay to compare DIABLO models that included/excluded the repeated measures experimental design. According to Fig. 1d–f, the area under curve (AUC) for the first component for lncRNA, mRNA, and miRNA was 1, suggesting a satisfactory result of the first models. In addition, the clustered image map (CIM) on the basis of the miRNA, mRNA and lncRNA selected on the first component well classified the tumor samples as well as normal samples (Fig. 2a). Meanwhile, we used a circos plot (Fig. 2b) to display selected features with different types. The side line showed the expression levels in each phenotypic group. The farther away from the center of the circle, the lower expression level of the phenotype. Here, we focused on the XIST/miR-140-5p/*ORC1* axis in CESC, and their target relationships were listed in Fig. 2c.

### LncRNA XIST is up-regulated in cervical cancer

Firstly, we divided 30 patients into two groups in accordance with the average XIST expression level (high XIST group: XIST expression level > average; low XIST group: XIST expression level ≤ average) to investigate the significance of XIST in cervical cancer. Our data indicated that XIST expression in cervical cancer tissues was statistically correlated with larger tumor size, advanced FIGO stage, well tumor differentiation and lymphatic metastasis (Table 1). However, there showed no dramatic correlation of XIST expression with some other clinical features such as age, pathological type (Table 1). Then, we explored XIST expression levels in tissue samples and cell lines through qRT-PCR. The results turned out that XIST expression level in cervical cancer tissues was dramatically higher than that in adjacent tissues (Fig. 2d). Additionally, when compared with human cervical epithelial cell line HcerEpic, XIST expression was ubiquitously increased in four cervical cancer cell lines (CaSki, Hela, C33A, SiHa) (Fig. 2e). In a nutshell, our findings documented that XIST may play a vital role in cervical cancer. Since XIST showed the highest expression in Hela and C33A cells, these two cell lines were selected for further study.

### LncRNA XIST inhibition suppressed cell proliferation in vitro

**Fig. 3:**
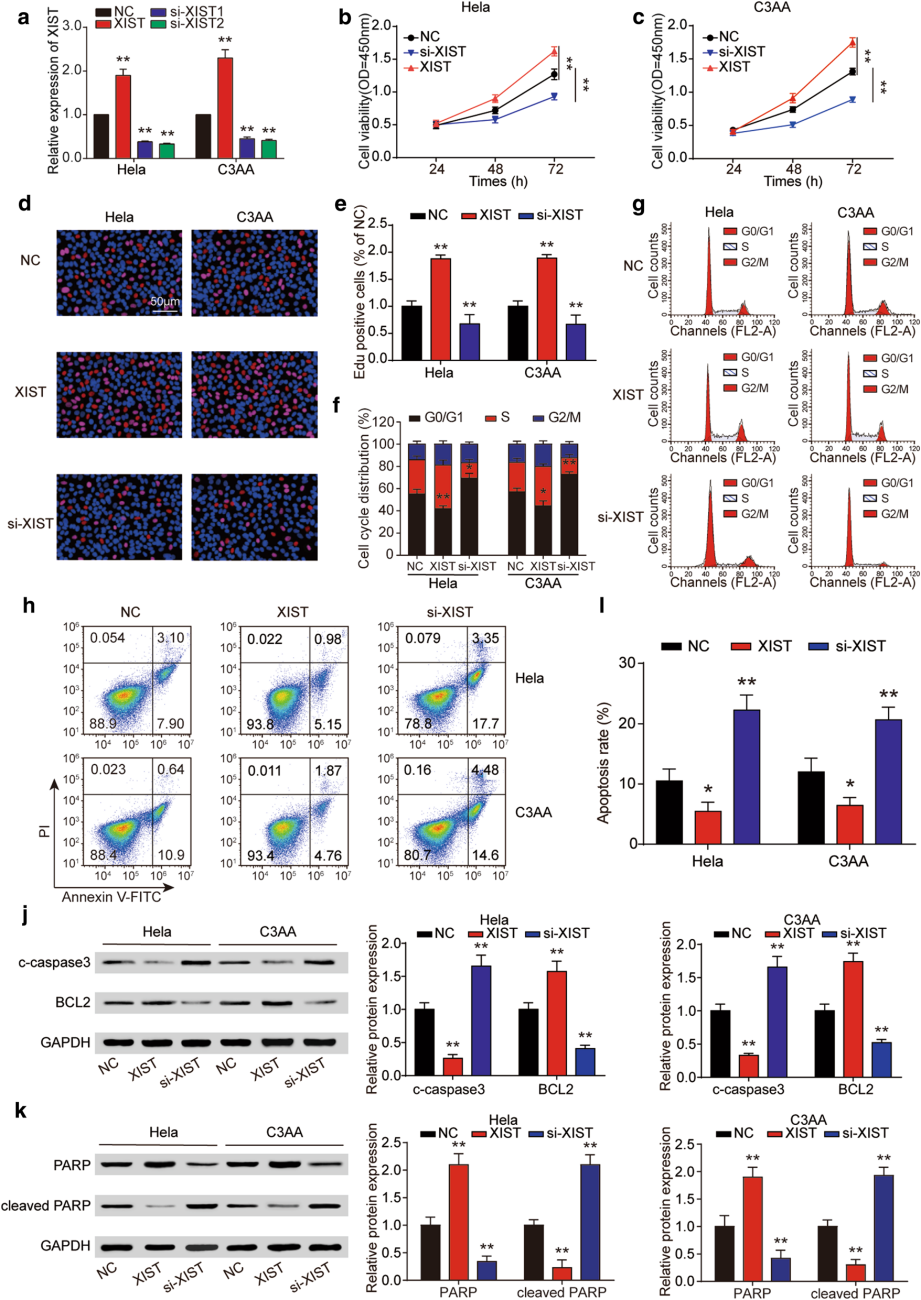
LncRNA XIST knockdown suppressed Hela and C3AA cell proliferation in vitro. **a** Relative expression of XIST after Hela and C3AA cells transfected with XIST, si-XIST or empty vector. It showed that the inhibition effect of si-XIST2 was obvious. The positive effect of XIST on cell proliferation of Hela and C3AA cells was detected by CCK-8 assay (**b**, **c**) and EdU assay (**d**, **e**). **f**, **g** Effect of XIST on cell cycle of Hela and C3AA cells was determined with flow cytometry. The results revealed that downregulation of XIST blocked cell cycle in G0 phase. **h**, **i** Overexpression of XIST led to the decreased cell apoptosis rate of Hela and C3AA cells detected by flow cytometry. **j**, **k** Hela and C3AA cells were transfected with XIST and si-XIST and subject to western blot analysis using antibodies against c-caspase3, Bcl-2, total PARP, cleaved PARP with GAPDH as a loading control. *P < 0.05, **P < 0.01, compared with NC

To make investigation on the effects of XIST on cancer cell proliferation, the XIST expression was elevated or reduced by transfecting pcDNA3.1-XIST or siRNAs specifically targeting XIST (si-XIST-1, si-XIST-2). QRT-PCR revealed that XIST expression was obviously up-regulated or down-regulated in Hela and C33A cells transfected with XIST or si-XIST compared with NC group (Fig. 3a), since the effect of si-XIST-2 was more obvious, the next experiment only used it to suppress the expression of XIST. And CCK-8 assay suggested that XIST significantly promoted cell proliferation both in Hela and C33A cell lines compared to NC group, which were reversed by the XIST downregulation (Fig. 3b, c). The EdU assay further confirmed the promoting effect of XIST on Hela and C33A cells proliferation (Fig. 3d, e). Besides the flow cytometry results demonstrated that cell cycle was blocked in G0 phase by si-XIST (Fig. 3f, g), whereas the apoptosis cells were increased in cervical cancer cells transfected with si-XIST compared to NC group (Fig. 3h, i). Furthermore, we measured the expression status of cleaved caspase3 (c-caspase3), B-cell lymphoma-2 (Bcl-2), total ploy ADP-ribose polymerase (PARP) and cleaved PARP. The results of western blot showed si-XIST induced significant accumulation of c-caspase3 and cleaved PARP whereas it decreased the expression of Bcl-2, proving that cell apoptosis was obviously promoted after transfection of si-XIST (Fig. 3j, k).

### LncRNA XIST inhibition suppressed cell proliferation in vivo

**Fig. 4:**
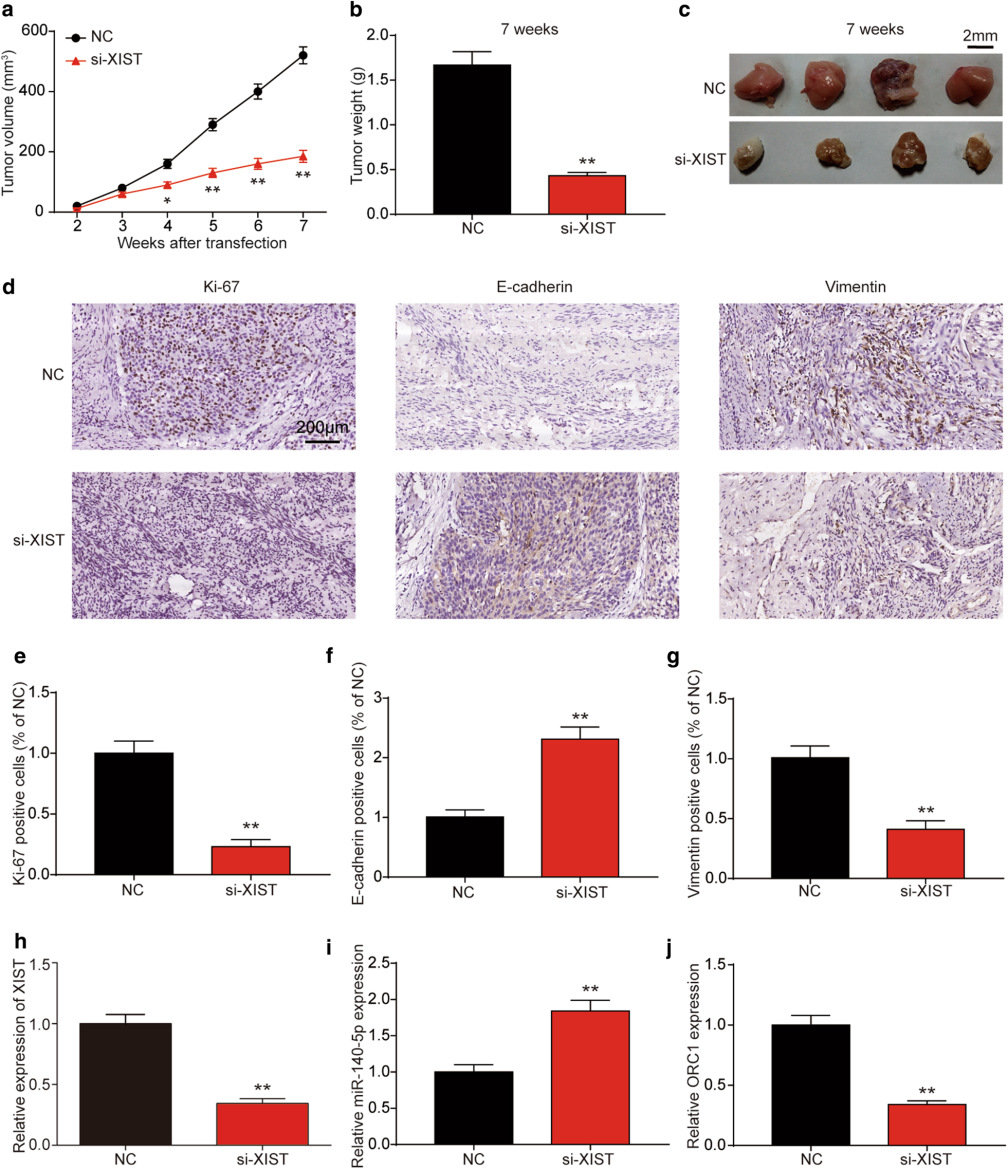
LncRNA XIST inhibition suppressed tumor growth in vivo. **a** Tumor growth curves determined after injection of Hela cells transfected with si-XIST or RNAi vector. The tumor volume was calculated every 7 days from 2 to 7 weeks. **b** Tumor weight of nude mouse was measured at the end of 49 days. **c** Photographs of tumors excised 7 weeks after si-XIST transfected Hela cells injected into nude mice. It was indicated that si-XIST inhibited tumor growth. **d**–**g** Photomicrograph showing immunohistochemical staining for Ki-67, E-cadherin and vimentin. Bar = 200 μm. The RNA expression of XIST (**h**), miR-140-5p (**i**) and *ORC1* (**j**) were determined by qRT-PCR after 7 weeks. Si-XIST increased the levels of E-cadherin and miR-140-5p, but decreased Ki-67, vimentin, XIST and ORC1 expression levels. *P < 0.05, **P < 0.01, compared with control

Si-XIST vector transfected Hela cells were inoculated into the mice to confirm the XIST effects on cervical cancer tumorigenesis. Xenograft tumors developed by all mice in injection site and we measure the volume of tumor every 7 days after transfection, as shown in Fig. 4a, tumor growth in si-XIST group was significantly slower than that in NC group. Furthermore, the average tumor weight in the si-XIST group was obviously lower than that in NC group as well (Fig. 4b, c). Furthermore, the results of immunohistochemical staining manifested that cells expressed Ki-67 and vimentin in tumor tissue of si-XIST group was obviously less than that of NC group, while E-cadherin was increased (Fig. 4d–g), indicating that the downregulation of XIST could inhibit cancer cell proliferation and EMT process in vivo. Furthermore, we detected the XIST, miR-140-5p and *ORC1* expression in xenograft tumors. Results showed that XIST and *ORC1* expression was significantly down-regulated in si-XIST injection group compared to control group after 7 weeks, while miR-140-5p expression was conversed (Fig. 4h, i). These results suggested that inhibition of XIST could significantly suppress proliferation capacity of cervical cancer in vivo, and these effects might be achieved through miR-140-5p and *ORC1*.

### XIST could regulate miR-140-5p by targeting it

**Fig. 5:**
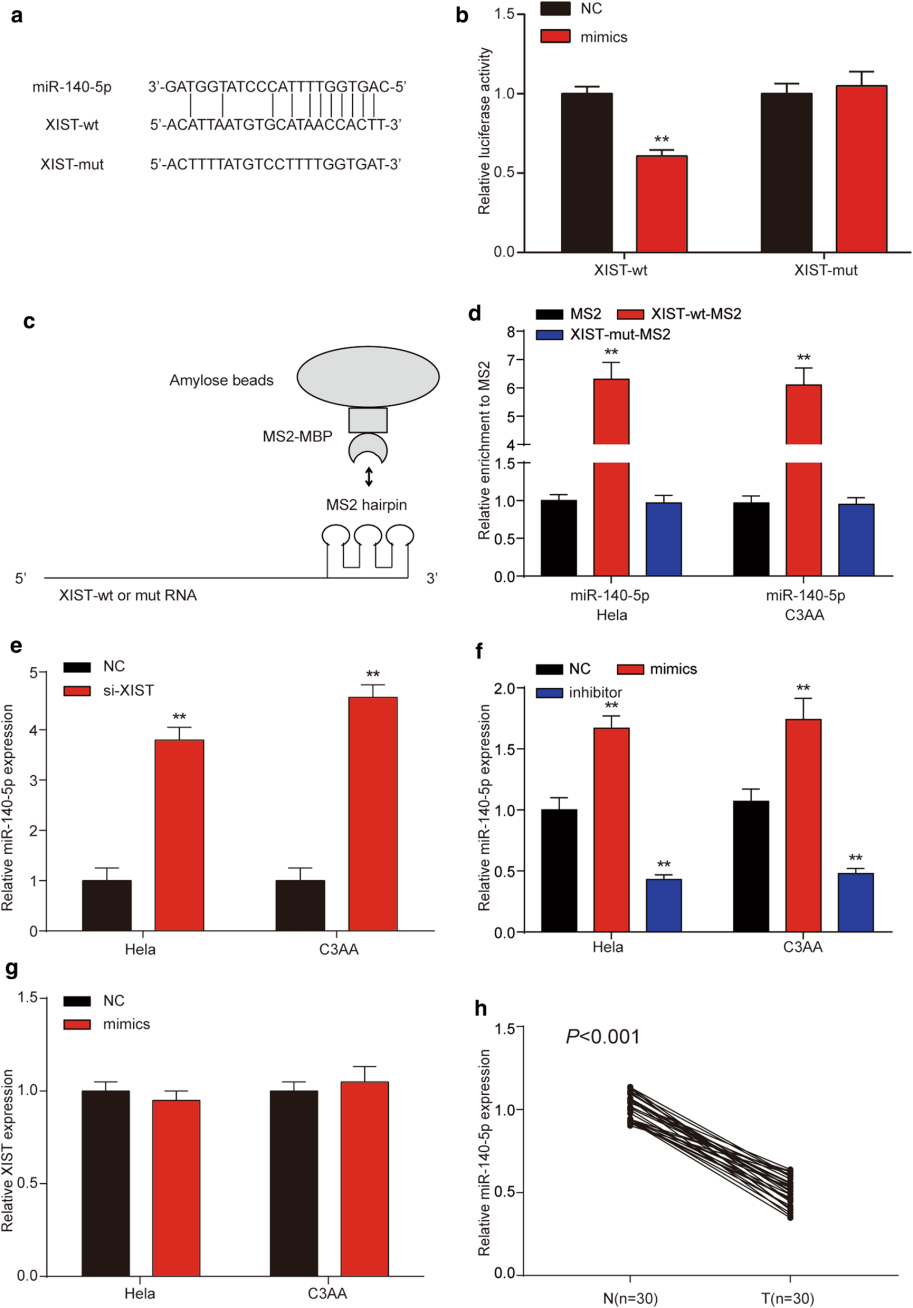
LncRNA XIST directly interacted with miR-140-5p. **a** Sequence alignment of miR-140-5p with the putative binding sites with in the wild-type regions of XIST. **b** Targeting relationship between miR-140-5p and *ORC1* in Hela cells was confirmed by dual-luciferase reporter assay. The luciferase activity was significantly reduced after co-transfection with miR-140-5p mimics and XIST-wt. **c**, **d** MS2-RIP followed by miRNA RT-PCR to detect endogenous miR-140-5p associated with the MS2-tagged XIST transcript. **e** QRT-PCR analysis of miR-140-5p expression in Hela and C3AA cells transfected with si-XIST or si-NC. **f** Relative expression of miR-140-5p in Hela and C3AA cells transfected with miR-140-5p mimics or inhibitor was quantified by qRT-PCR, which showed miR-140-5p was upregulated by miR-140-5p mimics and downregulated by miR-140-5p inhibitor. **g** XIST expression in Hela and C3AA cells was no change when the cells were transfected with miR-140-5p inhibitor or miR-NC. **h** The expression of miR-140-5p was determined by qRT-PCR in 30 pairs of cervical cancer tissues (T) compared with adjacent non-tumor tissues (N). It was showed that miR-140-5p was downregulated in cervical cancer tissues. “n” indicates sample number. **P < 0.01, ***P < 0.001, compared with miR-NC/MS2/si-NC/NC

To examine whether XIST has a targeting relationship with miR-140-5p, prediction of target sites between miR-140-5p and XIST was performed by the online software starBasev2.0 (Fig. 5a). Dual-luciferase reporter assay was practiced for the exploration of whether miR-140-5p functioned as a target of XIST. We discovered luciferase activity was remarkably decreased with the cell transfection of miR-140-5p mimics and XIST-wt rather than co-transfection of NC and XIST-wt, meanwhile, cells transfection of miR-140-5p mimics and XIST-mut had no effects on luciferase activity (Fig. 5b). Afterwards, RNA pull-down analysis was implemented to detect endogenous XIST associated miRNAs to further prove the direct interaction between XIST and miR-140-5p. QRT-PCR was adopted to analyze the precipitated miRNAs. We discovered that MS2-tagged wild-type XIST (XIST-wt-MS2) was remarkably enriched for miR-140-5p in Hela and C33A cells compared to the empty vector and XIST with a mutation in the miR-140-5p binding site (XIST-mut-MS2) (Fig. 5c, d). These discoveries indicated that the XIST worked as a ceRNA by sponging miR-140-5p. In addition, we found that si-XIST and miR-140-5p mimics led to significantly increased expression of miR-140-5p and miR-140-5p inhibitor could decrease the expression of miR-140-5p (Fig. 5e, f). However, up-regulated expression of miR-140-5p failed to affect XIST expression (Fig. 5g). Thus, the results showed that XIST had a negative regulation miR-140-5p.

### MiR-140-5p could suppress cervical cancer cells proliferation and induced cell apoptosis

**Fig. 6:**
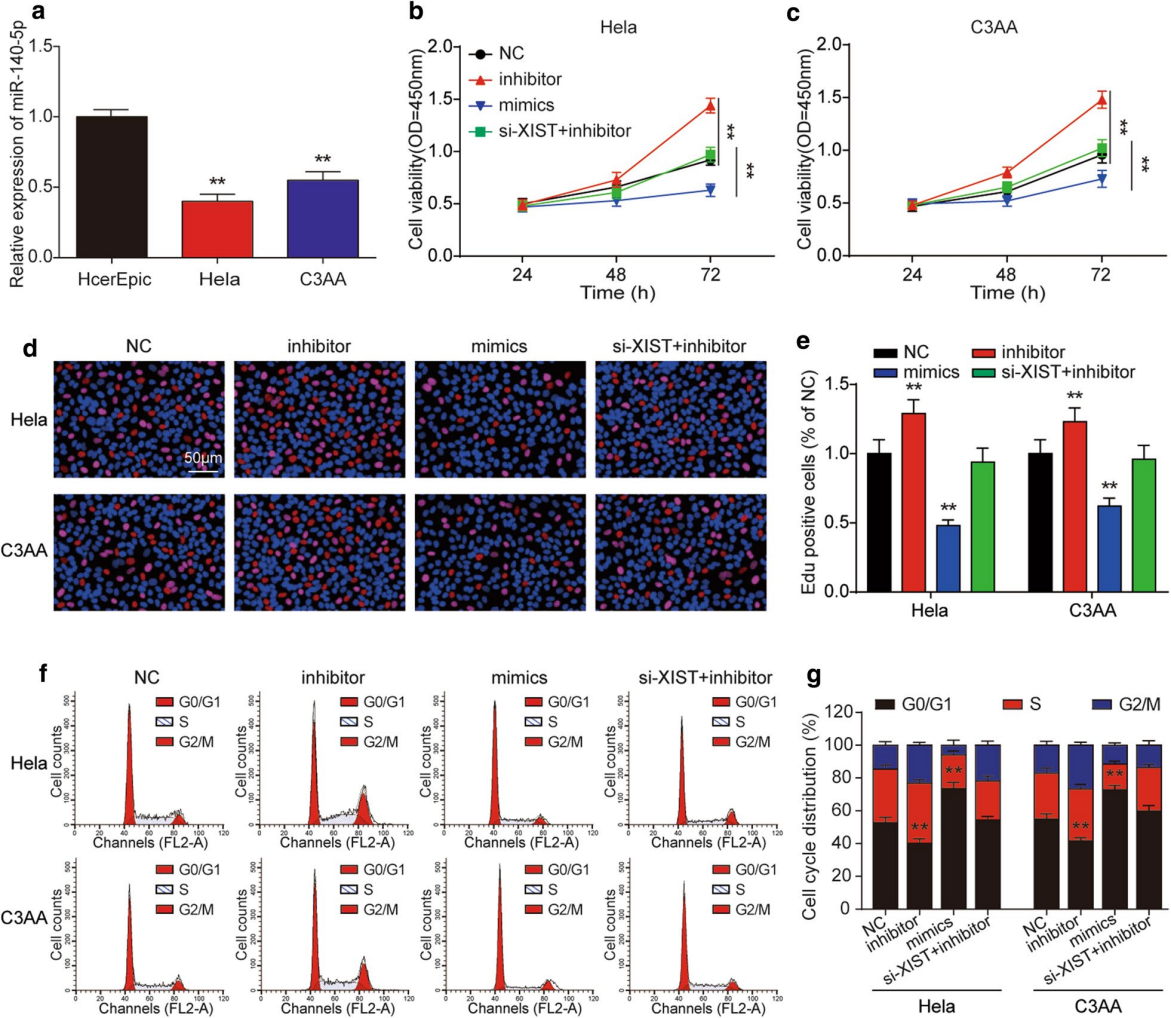
LncRNA XIST affected cervical cancer cell proliferation and cell cycle through miR-140-5p. **a** The expression of miR-140-5p was examined by qRT-PCR in two cervical cancer cell lines (HeLa, C3AA) and human cervical epithelial cell line HcerEpic. The inhibition effect of miR-140-5p on cell proliferation of Hela and C3AA cells was detected by CCK-8 assay (**b**, **c**) and EdU assay (**d**, **e**). **f**, **g** MiR-140-5p was verified to suppress cell cycle of Hela and C3AA cells using flow cytometry. **P < 0.01, compared with NC

**Fig. 7:**
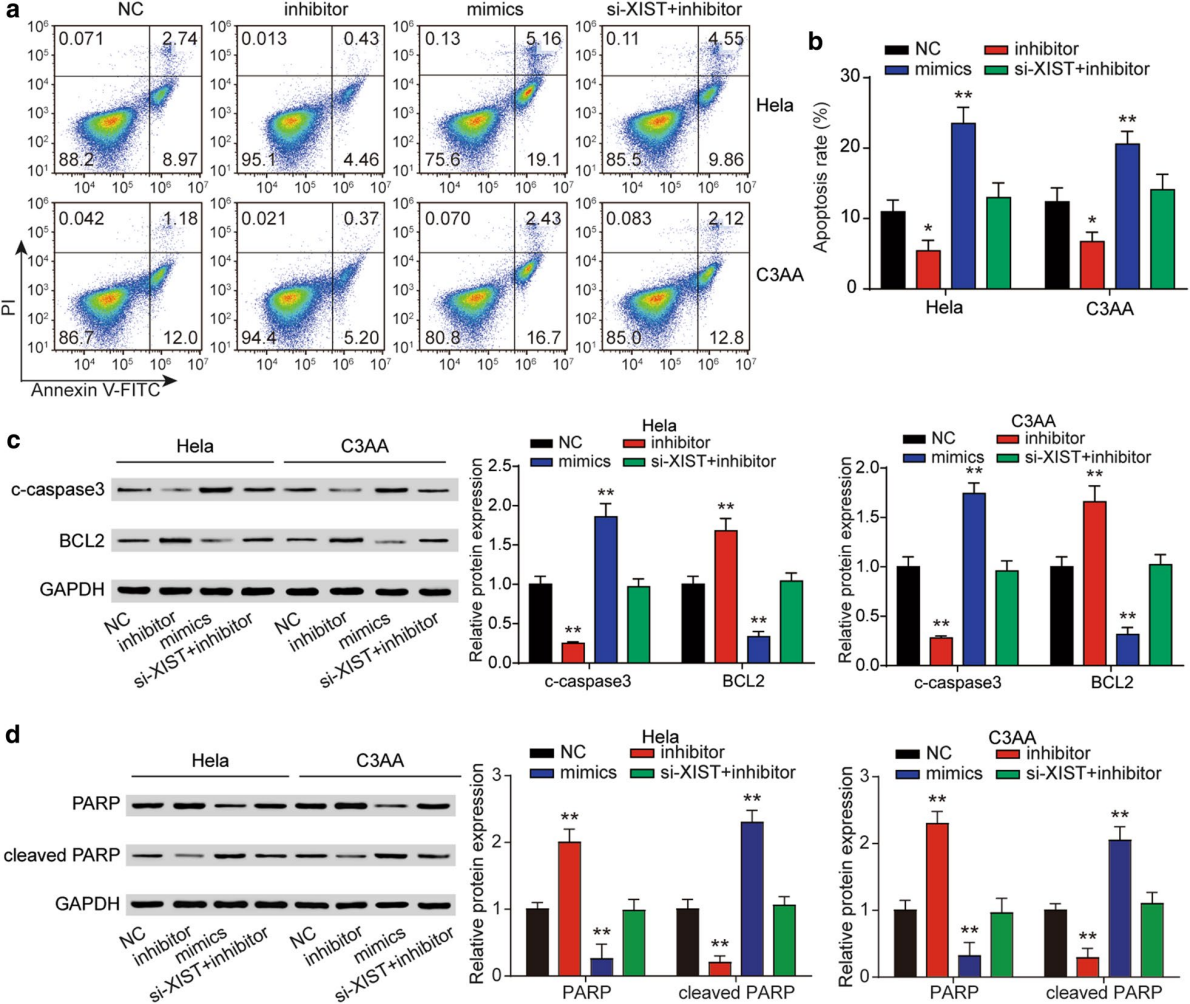
LncRNA XIST affected cervical cancer cell apoptosis through miR-140-5p. **a**, **b** MiR-140-5p induced Hela and C3AA cells apoptosis, which was detected by flow cytometry. **c**, **d** Hela and C3AA cells were transfected with miR-140-5p mimics, inhibitor or inhibitor + si-XIST and subject to western blot analysis using antibodies against c-caspase3, Bcl-2, total PARP, cleaved PARP with GAPDH as a loading control. *P < 0.05, **P < 0.01, compared with NC

We further determined the expression of miR-140-5p in tissues samples and cell lines. As predicted, miR-140-5p expression was decreased in tumor tissues samples and cancer cell lines (Figs. 5h, 6a). Subsequently, we researched the miR-140-5p effects on the cell proliferation of cervical cancer. Both the results of CCK-8 and EdU assay showed that transfection of miR-140-5p inhibitor significantly promoted cell proliferation both in Hela and C33A cells (Fig. 6b–e). In the Fig. 6f, g, the flow cytometry results manifested that cell cycle was accelerated after inhibiting miR-140-5p. Cell apoptosis analysis indicated that miR-140-5p could elevate cancer cell apoptotic rate (Fig. 7a, b). Furthermore, miR-140-5p induced significant accumulation of c-caspase3 and cleaved PARP whereas it decreased the expression of Bcl-2 (Fig. 7c, d). Besides, we found that si-XIST could offset the effects of miR-140-5p inhibitor in all of the above experiments which suggested that XIST affected cell proliferation in cervical cancer through regulating miR-140-5p.

### MiR-140-5p could regulate *ORC1* by targeting it

**Fig. 8:**
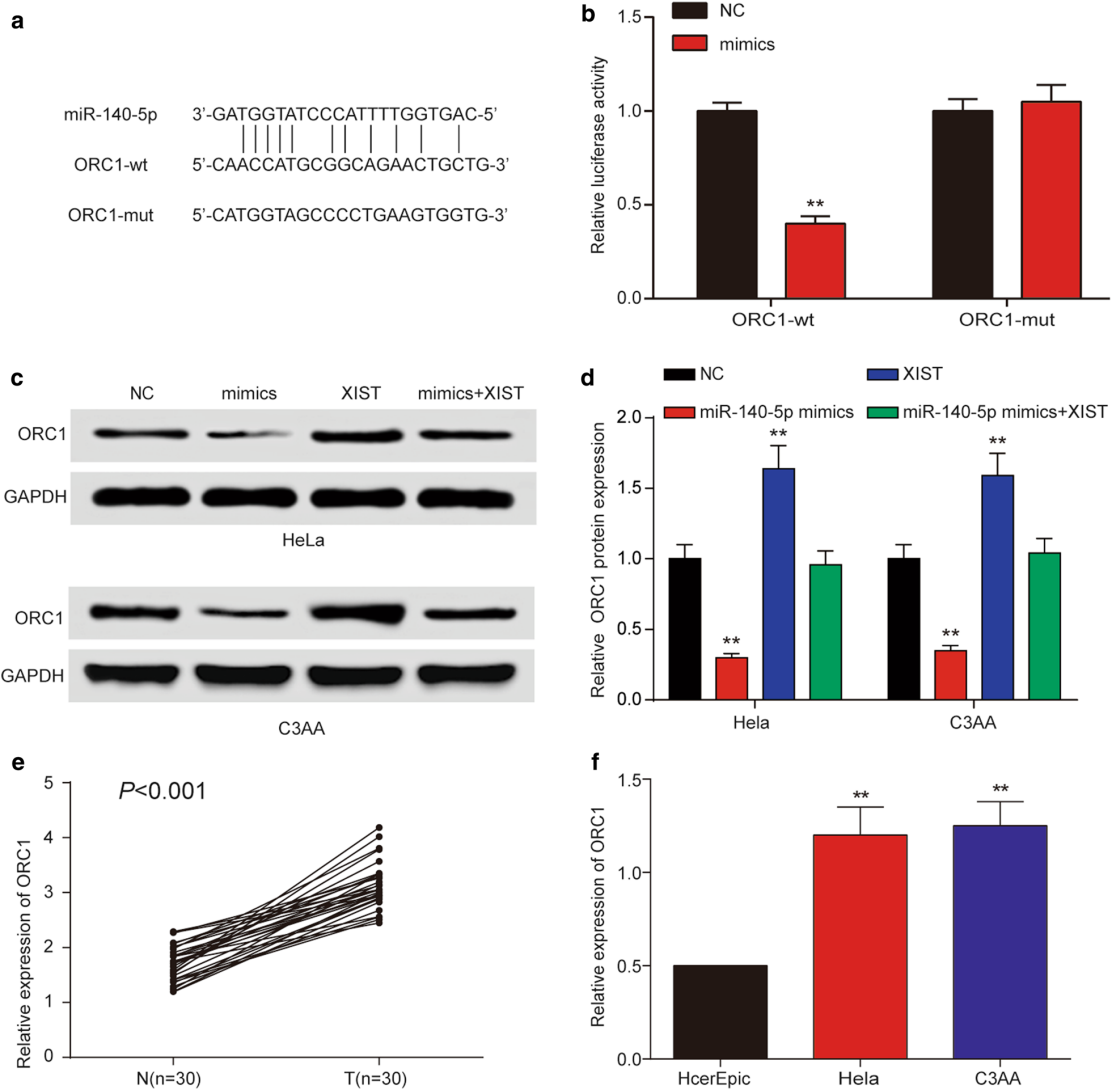
*ORC1* was a target of miR-140-5p and is suppressed by XIST inhibition. **a** Sequence alignment of miR-140-5p with the putative binding sites with in the wild-type regions of *ORC1*. **b** Targeting relationship between miR-140-5p and *ORC1* in Hela cells was confirmed by dual-luciferase reporter assay. **c** Expression of ORC1 was determined by western blot in Hela and C3AA cells transfected with miR-140-5p mimics or XIST or miR-140-5p mimics + XIST. **d** Quantitation of the *ORC1*/GRAPH ratios in Hela and C3AA cells. The western blot and qRT-PCR results manifested that XIST promoted *ORC1* expression which was inhibited by miR-140-5p. **e** The expression of *ORC1* was observably upregulated in 30 pairs of cervical cancer tissues (T) compared with adjacent non-tumor tissues (N) determined by qRT-PCR. “n” indicates sample number. **f** The expression of ORTC1 was examined by qRT-PCR in three cervical cancer cell lines (HeLa, C3AA) and human cervical epithelial cell line HcerEpic **P < 0.01, compared with miR-NC/NC/HcerEpic

Starbase was then applied used to predict target genes for miR-140-5p, and *ORC1* was one of the best candidates (Fig. 8a). Then, we constructed luciferase reporter assay, the result revealed that miR-140-5p overexpression significantly decreased the luciferase activity of the *ORC1*-*wt*, but not the *ORC1*-*mut* in 293T cells which confirmed the targeting relationship between miR-140-5p and *ORC1* (Fig. 8b). To further validate the *ORC1* regulation by miR-140-5p and XIST, we examined the expression of *ORC1* when miR-140-5p or XIST was overexpressed within Hela and C33A cells. Our results showed that increased miR-140-5p expression dramatically decreased the *ORC1* levels. The findings demonstrated that *ORC1* was a target gene of miR-140-5p. Meanwhile, the data of us indicated that XIST overexpression elevated the levels of *ORC1*. Furthermore, we found that XIST was no longer able to increase *ORC1* expression when miR-140-5p mimics was co-transfected (Fig. 8c, d, *P *< 0.05). QRT-PCR results also indicated that *ORC1* expression was ubiquitously increased in cervical cancer tissues and cell lines (HeLa and C33A) (Fig. 8e, f).

### *ORC1* could promote the progression of cervical cancer

**Fig. 9:**
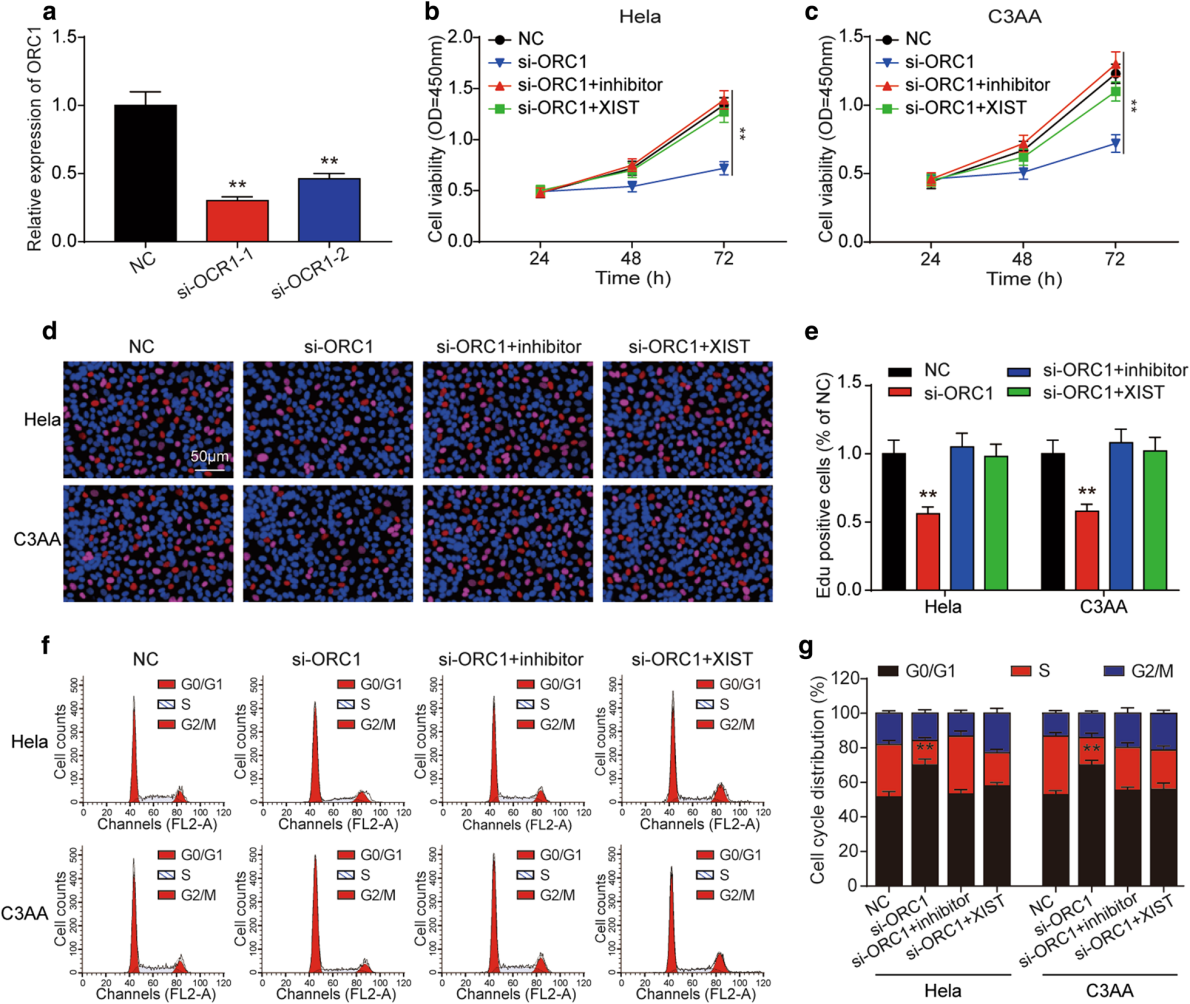
*ORC1* was required for the XIST function in cervical cancer cell cycle. **a** Relative expression of *ORC1* after Hela and C3AA cells transfected with si-*ORC1* or empty vector. The negative effect of *ORC1* downregulation on cell proliferation of Hela and C3AA cells was detected by CCK-8 assay (**b**, **c**) and EdU assay (**d**, **e**). **f**, **g** It was determined by flow cytometry that *ORC1* downregulation had a suppressed effect on cell cycle of Hela and C3AA cells. And the suppressed effect was offset by miR-140-5p inhibitor or upregulation of XIST. **P < 0.01, compared with NC

**Fig. 10:**
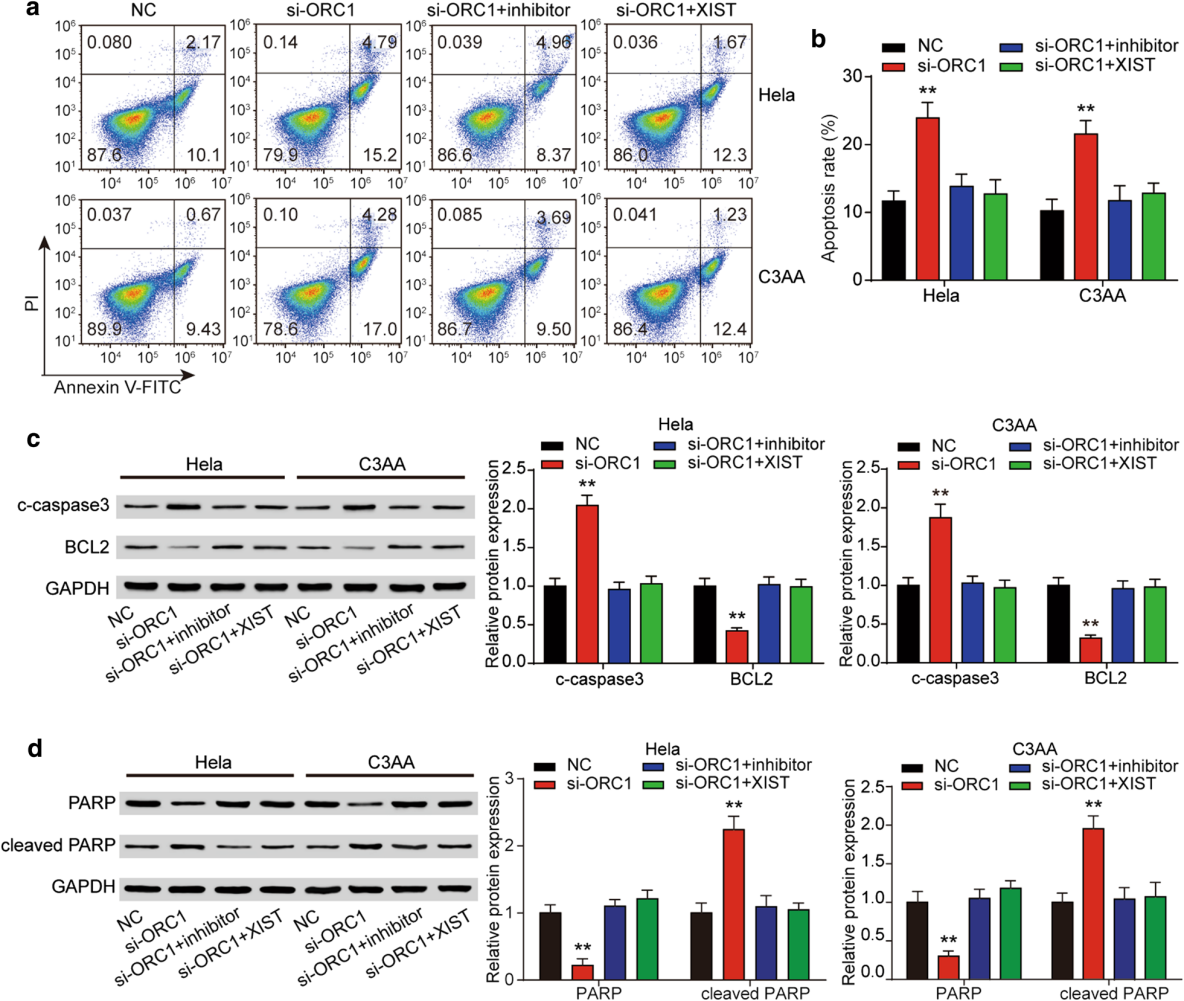
*ORC1* was required for the XIST function in cervical cancer cell apoptosis. **a**, **b** Effect of *ORC1* knock-down on cell apoptosis of Hela and C3AA cells was detected by flow cytometry. **c**, **d** C-caspase3, Bcl-2, total PARP, cleaved PARP protein expression in Hela and C3AA cell transfected si-*ORC1*alone or with miR-140-5p mimics or XIST. **P < 0.01, compared with NC

To further understand the role of *ORC1* in cervical cancer, we designed siRNAs specifically targeting *ORC1* (si-*ORC1*-1, si-*ORC1*-2), and the result of qRT-PCR showed si-*ORC1*-2 had the best effect for knockdown the expression *ORC1*, so it was used in the next experiments (Fig. 9a). Then, the cell proliferation was determined by CCK-8 assay, and the result showed knockdown the expression of *ORC1* could decrease the cell proliferation while miR-140-5p inhibitor and XIST could offset the effect of si-*ORC1* (Fig. 9b, c). EdU assay results in the Fig. 9d, e were similar with the CCK-8 assay in the Fig. 9b, c, which was demonstrated that *ORC1* suppressed the cervical cancer proliferation. And the results of cell apoptosis analysis were consistent with the previous experiments, si-*ORC1* could significantly promote the apoptosis rate of cervical cancer cells, and miR-140-5p inhibitor and XIST could offset the role of si-*ORC1* (Fig. 10a, b). Besides, the results of western blot showed si-*ORC1* could upregulate the expression of c-caspase3 and cleaved PARP and reduce the expression of Bcl-2, which further explained the effect of *ORC1* on the apoptosis of cervical cancer cells (Fig. 10c, d).

## Discussion

We found an evident increase of XIST expression in cervical cancer, and the downregulation of XIST inhibited cell proliferation and cell cycle as well as induced cell apoptosis in the present study. We further discovered the targeting relationship between XIST and miR-140-5p, and miR-140-5p and *ORC1*. The cells experiments proved that XIST could affect *ORC1* expression by regulating miR-140-5p, and the further experiments determined the role of XIST/miR-140-5p/*ORC1* axis in cervical cancer.

Recently, XIST was demonstrated to accelerate cervical cancer progression in vitro, including cell proliferation, apoptosis, invasion [25]. Likewise, we found significant overexpression of XIST in cervical cancer tissues as well as cell lines (Hela and C33A), accelerated the cancer cell proliferation and suppressed cell apoptosis. As we also validated that knock-down of XIST could suppress tumor growth in vivo which further confirmed the effects of XIST on cervical cancer progression.

In our study, we conducted immunohistochemistry staining to determine the expression of Ki-67, E-cadherin and vimentin, the results showed that Ki-67 and vimentin was decreased in cervical cancer tissues while E-cadherin was increased when we knockdown XIST. We knew epithelial-to-mesenchymal transition (EMT) that involved the loss of intercellular adhesion and acquisition of an invasive and migratory mesenchymal phenotype has been extensively relative to metastatic progression in a variety of cancers [26]. During EMT, cells lose epithelial characteristics such as the down-regulation of E-cadherin [27], which is one of the most commonly reported epithelial cell markers, and gain a mesenchymal phenotype with the high expression of mesenchymal proteins, including vimentin [28]. Therefore, the EMT process that is related to the expression of E-cadherin, vimentin which were generally used to demonstrate the ability of cancer cell metastasis and invasion [29]. All of these results above proved that XIST played a significant role in the cervical cancer metastatic.

It was widely known that the some lncRNAs acting as competing endogenous RNAs (ceRNAs) indirectly regulated mRNAs through shared miRNAs that represented a new layer of RNA crosstalk and played crucial roles in tumor progression [30]. For example, SNHG5 affects cell proliferation, metastasis and migration of colorectal cancer through regulating miR-132-3p/*CREB5* [31] and SNHG20 promotes the tumorigenesis of oral squamous cell carcinoma via targeting miR-197/*LIN28* axis [32]. In this study, we confirmed that XIST could regulate the expression of miR-140-5p. This was consistent with Tang et al.’s study, in which it was identified that XIST modulated the proliferation and apoptosis of lung cancer cells by regulating miR-140 [4].

Besides, we found the targeting relationship between miR-140-5p and *ORC1*, and related cell experiments further proved that XIST could regulate *ORC1* by targeting miR-140-5p to affect the progress of cervical cancer. Dong et al. [33] manifested that loss of miR-140 led to *PD*-*1* upregulation and then accelerated the cervical cancer cells proliferation and invasion. In Yao et al.’s study, miR-146b-3p could strongly promoted cervical cancer cells proliferation, migration and anchorage-independent and may be a potentially effective therapeutic target [34]. There have been already some studies that revealed *ORC1* upregulation resulted in DNA re-replication to trigger DNA damage response (DDR) in cancer cells, so some molecule arrested the cell cycle through inducing the accumulation of *ORC1* [35, 36]. In our study, we not only verified the blocking effect of *ORC1* downregulation on the cell cycle, but also demonstrated the inhibiting influence of *ORC1* on cell proliferation and the positive role in cell apoptosis.

Although our study showed the impacts of XIST on cervical cancer progression through miR-140-5p/*ORC1* axis, there were still some deficiencies remained to improve. For instance, the function of miR-140-5p and *ORC1* in vivo was not studied thoroughly. In addition, there may exist other miRNAs and possible mechanisms of XIST in cervical cancer, like miR-200a [25]. Therefore, more investigations need to be conducted to completely understand the mechanism of XIST in cervical cancer.

## Conclusions

In conclusion, our study proved that the XIST contributed to progression of cervical cancer cells by inhibiting miR-140-5p. Moreover, we carried a further exploration of the mechanism of XIST/miR-140-5p axis including the targeting gene *ORC1*, and provided a new therapeutic method for cervical cancer.

## Abbreviations

AUC: area under curve
CESC: cervical squamous cell carcinoma and endocervical adenocarcinoma
Bcl-2: B-cell lymphoma-2
CIM: clustered image map
CT: cervical thymus
EMEM: Eagle’s minimum essential medium
EMT: epithelial mesenchymal transition
MEM-EBSS: minimum essential medium-Earle’s balanced salts
XIST: X-inactive specific transcript
FBS: fetal bovine serum
HOX: homeobox
HVP: human papiloma virus
HSV-2: herpes simplex virus type 2
lncRNAs: long non-coding RNAs
ORC: origin recognition complex
RPMI: Park Memorial Institute
XIC: X inactivation center
ROC: receiver operating characteristic curve
SDS-PAGE: sodium dodecyl sulfate polyacrylamide gel electrophoresis
